# Impact of Emergent Physician Notifications from Mobile Cardiac Outpatient Telemetry on Patient Outcomes (The EP‐COT Trial)

**DOI:** 10.1111/jce.70354

**Published:** 2026-04-24

**Authors:** David Lin, Sanchit Kumar, Khurram Butt, Jake Klewer, Mahesh Balakrishnan, Alvaro Altamirano, Aaron Vigdor, Andrea Schell, Purvee Parikh, C. J. Grigoriadis, Manish Wadhwa, Mathew D. Hutchinson

**Affiliations:** ^1^ Department of Medicine, Cardiovascular Division, Electrophysiology Section Hospital of the University of Pennsylvania Philadelphia Pennsylvania USA; ^2^ Department of Medicine, Cardiovascular Division, Electrophysiology Section Banner‐University Medical Center, Sarver Heart Center, University of Arizona College of Medicine Tucson Arizona USA; ^3^ Philips Ambulatory Monitoring and Diagnostics San Diego California USA

**Keywords:** arrhythmia, emergent notification, remote monitoring, telemetry

## Abstract

**Introduction:**

Mobile ambulatory cardiac telemetry (MCOT) use has increased over time; however, data on the prevalence and the impact of emergent arrhythmia notifications with MCOT remains unclear. We sought to determine the prevalence and clinical impact of emergent arrhythmia events in patients undergoing MCOT monitoring. We also analyzed the efficiency of the emergent notification process.

**Methods:**

We analyzed 8404 consecutive patients from two centers who were prescribed Philips MCOT (K153473) over a 28‐month period (September 2018–January 2021). Participants meeting emergent notification criteria were included. The primary outcome was any unscheduled provider intervention after the emergent notification. We also analyzed several time domains of the provider notification process.

**Results:**

A total of 122 patients (1.45%) satisfied emergent notification criteria during the study period. The median notification time from arrhythmia onset to provider notification was 42 min. Physician review of the arrhythmia notifications showed agreement with the monitoring technician diagnosis in 102/122 (83.6%). An emergent notification resulted in an unscheduled follow‐up visit in 104/122 (85.2%) patients. Time from arrhythmia event to unscheduled follow up visit was < 24 h in 88/104 (84.6%), 24–72 h in 9/104 (8.7%) and > 72 h in 7/104 (6.7%). In 33 patients (27%), emergent notifications resulted in unscheduled interventions including: device implantation (24), ablation (8), and electrical cardioversion (4).

**Conclusions:**

Emergent arrhythmias events recorded during ambulatory telemetry monitoring resulted in unscheduled patient contact in 85% of cases and procedures in 27% of cases. The monitoring notification process was efficient, with median time from arrhythmia onset to provider notification of 42 min.

## 1 Introduction

The widespread availability of direct‐to‐consumer ambulatory ECG cardiac monitoring (CM) devices has created uncertainty regarding the optimal monitoring strategy for many arrhythmia patients. Conventional clinically‐utilized CM devices offer the advantages of continuous arrhythmia monitoring capabilities and datasets that are useful in diagnosing asymptomatic arrhythmias [1]. Selected ambulatory CM devices also provide wireless transmission capabilities that can identify clinically actionable rhythm disorders while the patient is being monitored, thereby avoiding the temporal delay in provider notification inherent in conventional CM devices. A prior study comparing over 78 000 patients demonstrated both improved diagnostic yield and shorter mean time to arrhythmia diagnosis in participants receiving a wireless connectivity device compared with a non‐wireless auto‐trigger device [1]. Provider notification criteria vary in commercially available wireless transmission CM devices. Provider notifications are generally categorized as urgent or emergent based upon rhythm severity. While urgent notification criteria are customizable by the provider, the emergent notification criteria are pre‐determined by the service provider and are typically non‐modifiable. After technician verification to confirm that emergent notification criteria were met, the result was then communicated to the ordering or on‐call provider irrespective of time of day. Neither the prevalence nor the clinical impact of device detected emergent notifications have been established. Furthermore, there is limited real‐world data regarding the temporal efficiency of provider notifications for emergent criteria detected by wireless transmission CM devices.

In this study we aimed to determine the prevalence and clinical impact of emergent notifications in patients receiving a wireless transmission CM device.

## 2 Methods

### 2.1 Patients

We conducted a retrospective study that analyzed all patients prescribed the Philips Mobile Cardiac Telemetry (MCOT) (Malvern, PA) at two large university practices (Banner University Medical Center, Tucson, AZ and the Hospital of the University of Pennsylvania, Philadelphia, PA) between September 2018 and January 2021. All monitors were prescribed for standard clinical indications, and decisions regarding specific monitor use was at the discretion of the ordering provider. All monitored patients ≥ 18yo were included in the analysis. Ethics approval was provided by the Institutional Review Boards at both centers. Waivers of authorization for use and disclosure of protected health information were granted as the study met the criteria for exemption under 45 CFR § 46.104, category 4. The study complied with all U.S. ethical standards and federal regulations.

### 2.2 Monitoring Process

Study participants received their CM either at clinical point‐of‐care or via mail for patients to self‐apply the monitoring patch. Participants were enrolled through the Philips Biotel monitoring company website at the time of monitor initiation, and all participants received both verbal and written education, as well as a video tutorial link, regarding monitor application and operation. The monitor was applied to the chest using an adhesive patch and was paired to a smartphone with cellular connectivity. The patients were instructed to wear the MCOT for the duration of the monitoring period, typically 30 days.

**Figure 1:**
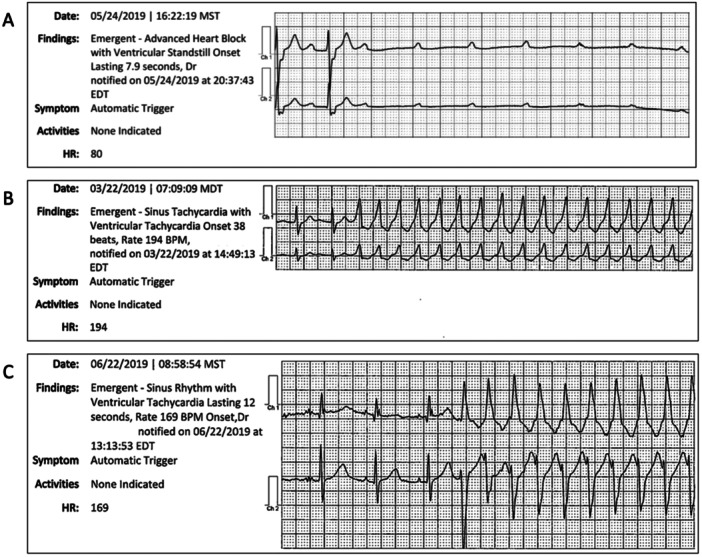
Three examples of emergent transmissions received from study participants. Panel A is taken from a 92‐year‐old man with recent syncope. The tracing reveals 7.9 s of asymptomatic AV block occurring in the afternoon while he was working in the yard. He underwent dual‐chamber pacemaker implantation. Panel B is taken from a 71‐year‐old man with CAD and a remote diagnosis of atrial fibrillation who had recent onset palpitations. The tracing reveals monomorphic ventricular tachycardia (VT). He had several subsequent alarms for a similar tachycardia lasting up to a minute and associated with palpitations. He was referred to the Emergency Department and was started on amiodarone with suppression of his VT. Panel C is taken from a 47‐year‐old man with rapid paroxysms of tachycardia. The tracing reveals onset of aberrant supraventricular tachycardia (deformation of third T wave in top lead, sharp intrinsicoid deflection) which was misdiagnosed as VT by the monitoring company. The patient underwent repeat EP testing where a concealed left posterolateral accessory pathway and inducible orthodromic AV reentry were identified.

Emergent events were detected by device‐based algorithms and transmitted automatically to the service provider. The time of the event was recorded on the monitoring device. The specific emergent notification criteria used by the MCOT CM are listed in Figure 1. Wireless transmission of emergent events required both proximity of the paired smartphone as well as the presence of an adequate cellular signal. The MCOT device (including sensor, phone, and app) could store up to 30 days of data without transmission in the event of loss of cellular coverage. Once received by the monitoring center, the emergent event was reviewed by a trained technician. If a true emergent event was adjudicated, then the technician attempted to contact the covering provider linked to the monitor via their preferred phone contact. The time that the monitoring technician contacted the provider was also recorded. Tracings from the emergent events were also sent via fax or email to the reading provider per the clinic's preference. The provider determined whether to contact the patient following the notification, and if needed, the time of patient contact was recorded from documentation in the medical record.

### 2.3 Outcomes

The primary study outcome was any unscheduled provider intervention after the emergent monitoring notification. Provider interventions were defined as: unscheduled phone contacts, new ambulatory or inpatient visits, or cardiac procedures. The nature and timing of the provider intervention were determined by retrospective chart review at each participating institution. We classified time domains from arrhythmia onset to patient contact: (1) < 24 h; (2) 24–72 h; and (3) > 72 h.

We also sought to gain insight into the efficiency of the monitoring process by analyzing three different time intervals: (1) time from arrhythmia event (AE) to receipt of transmission (RT) at monitoring company (AE‐RT); (2) time from receipt of transmission at monitoring company to provider notification (RT‐PN); and (3) time from provider notification to patient contact (PN‐PC). The times for AE, RT, and PN were provided by Philips for each event with PC based on electronic health record assessment.

The accuracy of the monitoring technician's diagnosis of each emergent tracing was also adjudicated by two independent electrophysiologists at each institution with reasons for common disagreements recorded as well.

### 2.4 Statistical Analysis

Categorical data are described as percentages and continuous data expressed as means(SD) or medians (IQR) as appropriate.

## 3 Results

In the study period, a total of *n* = 8404 participants underwent 30‐day MCOT monitoring over a 28‐month study period. The baseline characteristics of these participants are provided in Table 1. The most common indications for MCOT were atrial fibrillation (48%), palpitations (23%), syncope (3%), bradycardia (6%), and prior stroke (2%). A total of 122 (1.45%) participants had an Emergent Notification (*n* = 61 Banner Health, Tucson AZ, *n *= 61 Hospital of the University of Pennsylvania).

**Table 1:** Patient characteristics.

	Overall	UoA	HUP
*N*	122	61	61
Age (median, IQR)	63.2 (50, 73)	70 (55, 74)	61.3 (47.1, 69.3)
Female Gender	54 (44.3%)	28 (46%)	26 (43%)
Coronary artery disease	25 (20.5%)	14 (23%)	11 (18%)
Heart failure	45 (36.9%)	20 (33%)	25 (41%)
Diabetes mellitus	29 (23.8%)	13 (21%)	16 (26%)
Hypertension	65 (53.3%)	33 (54%)	32 (52%)
Cerebrovascular accident	18 (14.8%)	8 (13%)	10 (16%)
Monitoring indication:			
Atrial fibrillation/atrial futter	58 (48%)	28 (46%)	30 (64%)
Palpitations	28 (23%)	14 (23%)	14 (30%)
Syncope	4 (3%)	4 (7%)	—
Bradycardia (sinus pauses, AV block)	7 (6%)	4 (7%)	3 (6%)
Stroke	3 (2%)	3 (5%)	—

Abbreviations: HUP, Hospital of the University of Pennsylvania; UoA, University of Arizona.

Of the patients issued the alert, an unscheduled follow‐up visit occurred in 104 (85.2%) patients. This included a phone call (*n* = 96), office visit (*n* = 10) and ED/hospital visit (*n *= 20). Some of the patients received a phone call as well as office followup. The time from notification to the unscheduled follow‐up visit was < 24 h in 88/104 (84.6%), 24–72 h in 9/104 (8.7%) and > 72 h in 7/104 (6.7%). Overall, there were two patients where multiple attempts to contact were unsuccessful.

Emergent notification resulted in a subsequent intervention in 33 (27%) participants. This included device implantation in 24 (19.7%)–16 permanent pacemakers, 4 implanted cardioverter‐defibrillators, 4 implantable loop recorders with ablation in 8 (13%) and cardioversion in 4 (3.3%) participants.

Each of the emergent strips was independently adjudicated by two electrophysiologists at each study institution. There was 100% agreement between the two reviewers for each monitoring strip. There was agreement between the monitoring technician diagnosis and the adjudicated diagnosis in 102/122 (83.6%) participants. For the 20 strips where there was discrepancy with the technician diagnoses, the most common adjudicated findings were: supraventricular tachycardia (SVT) with aberrancy (7), artifact (3) and different duration of event(2).

The median time from arrhythmia event to monitoring center receipt of the transmission (AE‐RT) was 7 (IQR 3.5, 62.5) minutes. The median time from monitoring center receipt of the transmission to provider notification (RT‐PN) was 42 (IQR 18, 101) minutes. When contacted, the median time from provider notification to patient contact was 212.5 (IQR 18, 899.4) minutes.

## 4 Discussion

This study provides pertinent information to assess the efficiency and effectiveness of wireless telemetry technologies including diagnostic accuracy, timing of data transmission, and relevant patient outcomes. It showed that an Emergent Notification occurred in 1.45% patients monitored with MCOT, which resulted in an unscheduled patient encounter in 85% of cases. This notification also resulted in a cardiac procedure in 27% of cases. Additionally, the accuracy of technician diagnosis for the emergent tracings was ~84%, with a median time from arrhythmia event to provider notification of 42 min. The most common reasons for disagreement were SVT with aberrancy and artifacts misdiagnosed as ventricular tachycardia.

The original manuscript describing the use of ambulatory telemetry monitors in a large patient cohort was published nearly 20 years ago [2]. This included 100 patients with a high prevalence of existing arrhythmia diagnoses or periodic syncope and reported a high prevalence of asymptomatic arrhythmias that led to change in clinical care in 34% patients. Since then, use of ambulatory telemetry has increased substantially, with improvements in device design and wireless transmissibility contributing to more widespread utilization. In our more contemporary cohort, where ~2/3rd were monitored for palpitations or atrial fibrillation, emergent monitoring findings occurred in one of every 68 monitors applied which led to a clinic change in around a third of cases.

For selected patients with frequent, symptomatic arrhythmias, consideration of shorter‐term or on‐demand (i.e., wearable) technologies may be a more efficient and cost‐effective strategy. For patients with less frequent or minimally to asymptomatic arrhythmias, continuous monitoring devices capable of prolonged wear times substantially increases diagnostic yield. However, if the clinical suspicion for more malignant arrhythmias is moderately high (e.g., abrupt syncope, cryptogenic stroke, post TAVR new bundle branch block, etc), monitors with real time notification capability is essential. The EMBRACE trial randomized 572 patients with cryptogenic stroke to MCOT vs. 24‐h Holter monitoring and reported a 12.9% higher prevalence of AF (defined as > 30 s at 90 days) in the MCOT group (number needed to treat [NNT] 8) [3]. It is worth noting that in patients who did not have AF diagnosed on MCOT in EMBRACE, 85% of patients completed 3 weeks of monitoring and only 62% of patients completed all 4 weeks of prescribed monitoring; factors such as monitoring complexity and skin electrode hypersensitivity may contribute to patient non‐adherence with prolonged external cardiac monitoring. The transition from traditional lead‐based Holter monitors to patch‐based designs has increased patient satisfaction and adherence with monitoring, translating to improved diagnostic yield [4].

Contemporary arrhythmia management is often undertaken with extended duration monitoring, which entails extended wear Holter, MCOT or implantable cardiac monitors (ICM). The CRYSTAL AF trial, which ICM with short‐term Holter monitors in 441 cryptogenic stroke patients, reported a 10.4% increased yield with ICM at 12 months [5]. A recent report modelled the comparative cost of MCOT vs. ICM as a first‐line strategy in cryptogenic stroke patients. MCOT patients with a negative monitor at 30‐days were then modelled to proceed with ICM. The cost analysis estimated a 4.6% increased AF yield with MCOT compared to ILR, likely related to the MCOT's ability to detect short duration AF as compared to the ICM, which requires minimum of 2 min to satisfy detection criteria. MCOT as an initial strategy followed by ICM, as needed, represented a cost savings of $4 000 000 in the cohort of 1000 patients [6].

Our study found consistent and acceptable timing from the arrhythmia event to data transmission (7 min) and physician notification (42 min) with MCOT. This is compared to non‐wireless devices, which require offline analysis of data that leads to temporal delays in diagnosis and treatment, not uncommonly exceeding 4 weeks when including mail back, processing and interpretation of study.

Overall, we found a high degree of correlation between the preliminary technician findings and the adjudicated strip interpretations (~84%) with common reasons for disagreement being presence of supraventricular tachycardia with aberrancy and presence of artifact. As technology continues to improve and artificial intelligence is more broadly implemented as an additional layer of rhythm interpretation, the accuracy of the rhythm diagnosis should continue to improve.

The emergent notifications were clinically impactful in that they led to unscheduled visits and invasive interventions in a substantial proportion of participants (~85%).

Our study is strengthened by the large number of patients analyzed at two different centers with indications closely resembling those seen in contemporary clinical practice. We also looked at important clinical outcomes including procedures that resulted from the emergent event notifications. Utilization of two independent reviewers for confirmation of technician adjudicated findings also allowed for more definitive description of the accuracy of MCOT emergent notifications.

## 5 Limitations

Our study has certain limitations. Namely, cardiac monitoring technologies differ in arrhythmia detection and data transmission algorithms; therefore, the results of our trial are specific to Philips MCOT and may not be broadly applicable to other mobile telemetry vendors. Our study did not analyze demographic data or monitoring indications in patients who did not have emergent findings and it was therefore not possible to gain insight into specific populations at higher risk for these events. We also do not report monitoring adherence, which could have led to under‐reporting of emergent events due to a reduction in monitoring duration. Additionally, while participants could have multiple emergent events during monitoring, the current study only described the initial emergent event and clinical outcomes related to it. Finally, the use of 10 s pause as the criteria for notification is likely longer than what most providers might feel comfortable using. However, we intentionally chose this as a cutoff to limit the notifications to those who would be most likely to necessitate an intervention. It is possible that if the criteria for pause notification was 6 s as the cutoff, it could have resulted in an even higher rate of unscheduled patient visits.

## 6 Conclusions

Emergent notifications from ambulatory cardiac telemetry monitoring with Philips MCOT are efficient, accurate, and clinically impactful, resulting in unscheduled provider contact in 85% and cardiac procedures in 27% of cases.

## Disclosure

Authors (C.J.G, P.P., M.W.) are employees of Philips.

## Supporting information

Supporting File

## Data Availability

The authors have nothing to report.
