# Supplementary material for: Impact of Emergent Physician Notifications from Mobile Cardiac Outpatient Telemetry on Patient Outcomes (The EP‐COT Trial)
Source: J Cardiovasc Electrophysiol. 2026 Apr 24;37(6):1321–5. doi: 10.1111/jce.70354 (PMC13269860; doi:10.1111/jce.70354)
Supplement: Supplementary file 1 — Supporting File [file JCE-37-1321-s001.docx]

Supplementary Table 1: Emergent notification criteria

| Pause: All Events ≥ 10▒seconds |
| --- |
| Severe Tachycardia: ≥220 BPM,▒≥▒30▒seconds |
| Severe Bradycardia: < 20 BPM, ≥30▒seconds |
| Ventricular Fibrillation: All Events |
| Ventricular Tachycardia 100-149 bpm: ≥30 beats with symptoms |
| Ventricular Tachycardia 150-189 bpm: ≥10▒seconds (asymptomatic) OR Any symptomatic episode |
| Ventricular Tachycardia ≥190 bpm: ≥30 beats (asymptomatic) OR Any symptomatic episode |
